# Electrochemical Mechanism of Recovery of Nickel Metal from Waste Lithium Ion Batteries by Molten Salt Electrolysis

**DOI:** 10.3390/ma14226875

**Published:** 2021-11-15

**Authors:** Hui Li, Yutian Fu, Jinglong Liang, Chenxiao Li, Jing Wang, Hongyan Yan, Zongying Cai

**Affiliations:** College of Metallurgy and Energy, North China University of Science and Technology, Tangshan 063210, China; lh@ncst.edu.cn (H.L.); fu1849759929@163.com (Y.F.); lichenxiao@ncst.edu.cn (C.L.); xongmao9786@163.com (J.W.); yanhy@ncst.edu.cn (H.Y.); caizy@ncst.edu.cn (Z.C.)

**Keywords:** used lithium-ion batteries, recycling nickel, electrochemical reduction, constant current electrolysis

## Abstract

With the widespread use of lithium-ion batteries, the cumulative amount of used lithium-ion batteries is also increasing year by year. Since waste lithium-ion batteries contain a large amount of valuable metals, the recovery of valuable metals has become one of the current research hotspots. The research uses electrometallurgical technology, and the main methods used are cyclic voltammetry, square wave voltammetry, chronoamperometry and open circuit potential. The electrochemical reduction behavior of Ni^3*+*^ in NaCl-CaCl_2_ molten salt was studied, and the electrochemical reduction behavior was further verified by using a Mo cavity electrode. It is determined that the reduction process of Ni^3+^ in LiNiO_2_ is mainly divided into two steps: LiNiO_2_ → NiO → Ni. Through the analysis of electrolysis products under different conditions, when the current value of LiNiO_2_ is not less than 0.03 A, the electrolysis product after 10 h is metallic Ni. When the current reaches 0.07 A, the current efficiency is 77.9%, while the Li*^+^* in LiNiO_2_ is enriched in NaCl-CaCl_2_ molten salt. The method realizes the separation and extraction of the valuable metal Ni in the waste lithium-ion battery.

## 1. Introduction

China’s primary metal resources are gradually diminishing [1,2]. The recovery of valuable metals in waste lithium-ion batteries has major significance for the comprehensive utilization of waste resources [3,4].

Previous studies mainly adopted hydrometallurgical technology [5,6,7,8,9,10,11], which realized the recovery and utilization of Ni and other valuable metals through leaching, roasting and other processes. Although the method has a higher metal recovery and lower energy consumption, the entire process requires excessive acid-base solution and produces too much waste. Based on wet technology, the biological leaching method [12,13] has problems such as a long cycle and harsh microbial culture conditions. At present, molten salt electrolysis technology continues to develop, mainly through electro-chemical methods to control the reduction process to prepare metals or alloys.

Chen et al. [14,15,16,17,18] used CaCl_2_ melt to directly reduce TiO_2_, NiO, TiO, ZrO, Tb_4_O_7_, Fe_2_O_3_, etc., using a solid oxide or a mixture of two solid oxides as raw materials. The corresponding Ti metal, Cr metal, TiNi alloy, TiZr alloy and TbFe_2_ alloy were prepared, and the solid Cr_2_O_3_ electrolysis process was optimized in CaCl_2_ and NaCl-CaCl_2_ molten salt [19]. The feasibility of the melt’s electrolysis process in the preparation of metals or alloys has been confirmed in the above studies. For the extraction of valuable metals from waste lithium ions, Yin Huayi et al. [20] used Na_2_CO_3_-K_2_CO_3_ molten salt to successfully prepare Li and Co through the electrolysis of LiCoO_2_. Compared with hydrometallurgy, the molten salt electrolysis process does not use a strong acid solution, so it will not produce too much waste. At the same time, compared with large-scale and large-emission pyrometallurgy, molten salt electrolysis does not use reducing agents, so the cost is lower, the exhaust gas emissions are small, and it is environmentally friendly [20,21].

The NaCl-CaCl_2_ molten salt was selected to study the electrolysis behavior of LiNiO_2_. Using electrochemical testing methods of cyclic voltammetry, square wave voltammetry, chronoamperometry and open circuit potential, the reduction mechanism of LiNiO_2_ in the electrolysis process was analyzed, and metal Ni was successfully prepared by constant current electrolysis. The products at different stages in the reduction process were analyzed and the optimal reduction conditions were determined. The recycling process is simple, easy to operate, high in efficiency and does not introduce other impurities, and provides a new idea for the recycling of precious metals in waste lithium ion batteries.

## 2. Experimental Method

The electrochemical test was carried out using a three-electrode system. The platinum wire placed in the alumina tube (Φ = 4 mm) was used as the reference electrode, the Mo cavity hole electrode (2 mm × 1 mm × 30 mm) was used as the working electrode and the high-purity graphite sheet (99.99%, 20 mm × 5 mm × 100 mm) as the auxiliary electrode. First, the pure salt (NaCl, CaCl_2_) was placed in a DZF-6020 vacuum drying oven (Boxun, Shanghai, China), dried at a constant temperature of 220 °C for 600 min and weighed 150 g with a molar ratio of n_NaCl_:n_CaCl2_ = 1:1; it was then crushed and mixed evenly, and put into the corundum crucible. Then, the mixed salt was put in a vacuum drying oven and removed after drying at 250 °C for 12 h. The dried mixed salt was placed in a resistance heating furnace (Yunjie, Baotou, China) heated at 5 °C/min to 750 °C, then kept at a constant temperature for 120 min, and argon gas was passed through the entire process for protection. Next, the electrodes were inserted into the molten salt to test the electrochemical behavior of the Mo working electrode and the Mo cavity electrode. Then, 2 g of the LiNiO_2_ and Mo cavity electrode filled with LiNiO_2_ were respectively added to the melts, kept at a constant temperature for 240 min, and the three-electrode electrochemical test was performed again.

The electrolysis experiment was carried out with a two-electrode system. The 0.7 g sintered LiNiO_2_ flakes (about 15 mm in diameter and about 1 mm in thickness) were tightly wrapped with stainless steel mesh, and then fixed on a stainless steel rod as a cathode. High-purity graphite flakes (99.99%, 20 mm × 5 mm × 100 mm) were fixed on another stainless steel rod as an auxiliary electrode. The two electrodes were placed in the NaCl-CaCl_2_ molten salt at 750 °C; the electrolysis current range was 0.01–0.15 A and the electrolysis time was 0–10 h. After the electrolysis, the product was quenched and cleaned, and the sample was tested after drying.

The electrochemical test used Chenhua CHI660E (Chenhua, Shanghai, China) electrochemical workstation, and the Noran7X-ray diffractometer (Rigaku, Tokyo, Japan) was used to analyze the product.

## 3. Experimental Results and Discussion

### 3.1. The Electrochemical Behavior of Ni^3+^ on Mo Electrode

#### 3.1.1. Cyclic Voltammetry

**Figure 1:**
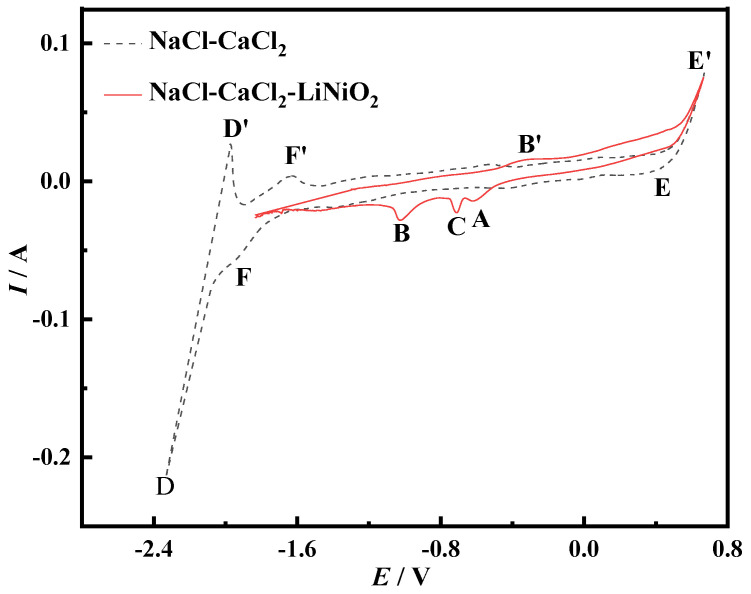
Cyclic voltammogram of Mo electrode before and after adding LiNiO_2_ to NaCl-CaCl_2_ molten salt (scanning speed: 0.25 V/s, *T* = 750 °C, reference electrode: Pt electrode).

Figure 1 shows the cyclic voltammetry test results of NaCl-CaCl_2_ molten salt and NaCl-CaCl_2_-LiNiO_2_ molten salt when the scanning speed is 0.25 V/s. The dotted line in the figure is the cyclic voltammetry curve of NaCl-CaCl_2_ molten salt. It can be seen that there are only the reduction peaks of Ca^2+^ and Na^+^ and the oxidation peaks of Ca and Na, indicating that the NaCl-CaCl_2_ molten salt system is between −1.68 V and 0.37 V. No oxidation–reduction reaction occurs, and this range can be used as the scanning range after adding LiNiO_2_ to the NaCl-CaCl_2_ molten salt.

The theoretical decomposition voltages of NaCl and CaCl_2_ at 1023 K are calculated from the data obtained from HSC Chemistry6.0 thermodynamic calculation software (Outokumpu, Finland). The theoretical decomposition voltages are −3.238 V and −3.325 V, respectively. Since the theoretical decomposition voltage difference between the two is relatively small, peak D and peak D′ are the overlapping reduction peak and oxidation peak of Na and Ca, respectively. Since Na is volatile, the peak D′ is smaller than the reduced peak D. Since CaCl_2_ also has a certain degree of water absorption in molten salt, the formation of CaO causes the peak F to be relatively gentle. The formation of the peak F′ is mainly due to the oxidation of the elemental Ca [22]. Peak E′ corresponds to the oxidation of Cl^−^. The generation of Cl_2_ changes the ion concentration around the molten salt and increases the reduction rate. The increase in the reduction rate increases the current to form peak E. The cyclic voltammetry curve shown by the solid line in the figure has multiple peaks, indicating that the electrochemical process of Ni^3+^ on the Mo electrode is a multi-step reduction process.

Konishi et al. [23] studied the process of preparing Fe-Dy alloy thin films by the molten salt electrochemical method. In this process, iron and dysprosium can form alloys. It can be seen from the electrochemical cyclic voltammetry curve that the reduction peak potential during the reaction is more positive than the deposition potential of dysprosium metal, so this peak is considered as an alloy peak. Kubota et al. [24] studied the formation mechanism of Co-Gd alloy films by regulating the electrochemical reaction process of molten salt. Since cobalt and gadolinium can form an alloy, it is judged that the peak corresponding to the reduction current in the cyclic voltammetry curve is the alloy peak. The objects of this study, Ni and Mo, will also form an alloy. When using a Mo electrode, there are three reduction peaks in the cyclic voltammetry curve of Ni. The peak potential of peak A is more positive than the deposition potential of Ni^3+^. It is judged that peak A is an alloy peak based on literature; there is no electron transfer and metal ion reduction in the process. Therefore, the reduction process of Ni^3+^ when using a Mo electrode is a two-step reduction.

**Figure 2:**
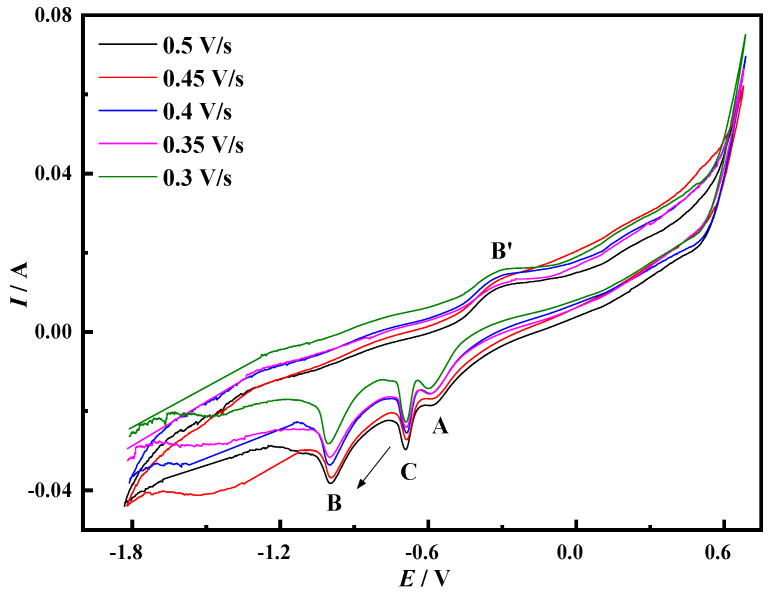
Cyclic voltammogram on Mo electrode in NaCl-CaCl_2_-LiNiO_2_ system (Scan speed: 0.3–0.5 V/s, *T* = 750 °C, reference electrode: Pt electrode).

The cyclic voltammetry curve results of the Mo electrode measured at different sweep speeds in the NaCl-CaCl_2_-LiNiO_2_ system at 750 °C are shown in Figure 2. Under certain sweep speed conditions, when the potential reverse scanning process reaches the first chemical reaction reduction potential, the current gradually increases with the change in the potential. With the continuous negative shift of the scanning potential, Ni^3+^ is gradually enriched at the cathode. In this process, the charge transfer speed is slow, which causes the current to not continue to increase, and finally forms the limiting current.

As the electrochemical reaction progresses, the reduction products at the cathode diffuse into the molten salt slowly, occupying the reduction sites at the cathode. The lower concentration of reactive ions reduces the number of charge transfers and reduces the current, eventually forming peak C. The electrolysis process is mainly controlled by diffusion. When the reduction potential of the second chemical reaction is reached, the reduction process changes from the charge transfer process to the diffusion process, and peak B is formed. During the forward sweep of the potential, the Ni elemental substance is re-oxidized to form the peak B′. It can be seen from Figure 2 that the peak potentials *E_pc_* and *E_pa_* of peaks B and B′ are affected by the scanning speed *v*. The increase in the scanning speed makes the electrochemical reaction speed faster, promotes the rapid transfer of electric charge and increases the limiting current.

The Ni^2+^ in the molten salt is transported to the surface of the cathode through liquid phase mass transfer, and electrons are obtained between the two-phase interface of the cathode and the molten salt and, finally, metallic Ni is formed on the surface of the cathode. In the above process, the formation speed of metal Ni is faster, and the speed of mass transfer and electron transfer is slow, which leads to the phenomenon of electrode polarization. This phenomenon causes the electrode potential to move in a direction deviating from the equilibrium potential, which is manifested in that the peak potential of peak B moves in the negative direction, and the peak potential of peak B′ moves in the positive direction. According to the characteristics of the cyclic voltammetry curve of peak B, it is judged that peak B corresponds to a quasi-reversible reaction. The peak current of peak C increases with the increase in the scanning speed, there is no corresponding oxidation peak and the electrode reaction is judged to be an irreversible reaction.

**Figure 3:**
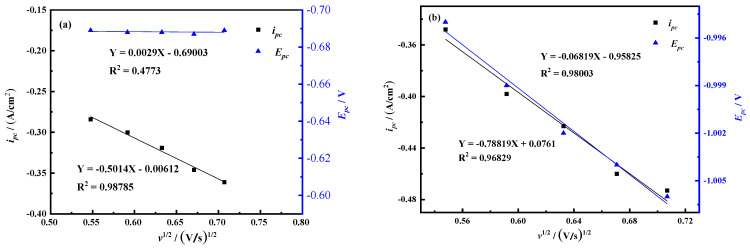
Curves of reduced peaks *i_pc_*-*v*^1/2^ and *E_pc_*-*v*^1/2^ (**a**) Peak C (**b**) Peak B (A = 0.08046 cm^2^).

In order to judge the control steps of the electrochemical reaction process, the *i_pc_*-*v*^1/2^ and *E_pc_*-*v*^1/2^ curves of peak C and peak B are drawn according to Figure 2, as shown in Figure 3. The current density value *i_pc_* of peak C has a linear relationship with the square root of the scan rate *v*^1/2^, and this process is mainly controlled by diffusion transfer. The current density value *i_pc_* and peak potential of peak B are linearly related to the square root of the scan rate *v*^1/2^, and the process is jointly controlled by diffusion and electron transfer.

#### 3.1.2. Square Wave Voltammetry

**Figure 4:**
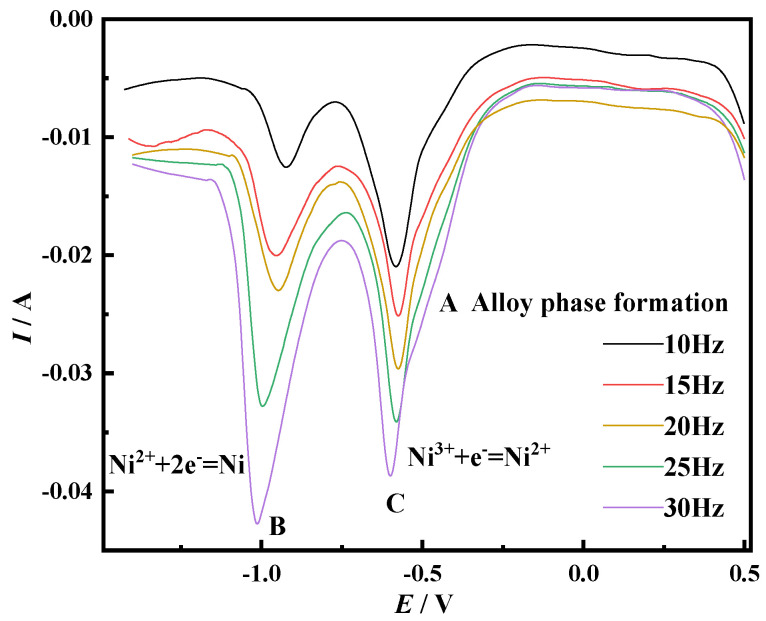
SWV diagram of the Mo electrode in the NaCl-CaCl_2_-LiNiO_2_ system (*f*: 10–30 Hz, *T* = 750 °C, reference electrode: Pt electrode, area = 0.08046 cm^2^).

In order to study the electrochemical reduction behavior of Ni^3+^ in the NaCl-CaCl_2_-LiNiO_2_ molten salt system, the square wave voltammetry with higher resolution and sensitivity was used to measure at different frequencies. This method can effectively suppress the capacitive background current that often occurs in cyclic voltammetry at relatively high scanning speeds. The selected test temperature is 750 °C, and the frequency is 10–30 Hz. The curve is shown in Figure 4.

There are three peaks A, C and B in the square wave voltammetry curve, and peak A is the alloy peak. The presence of peaks B and C indicates that Ni^3+^ forms a Ni elementary substance through a two-step electrochemical reduction process, which is consistent with the results of the cyclic voltammetry test.

In the square wave volt–ampere curve, the relationship between the half-width *W*_1/2_ and the number of transferred electrons *n* is expressed by Formula (1).

(1) $$W_{1/2}=3.52\times \frac{RT}{nF}$$

In the formula: R—molar gas constant (8.3145 J/mol/K), *T*—temperature (K), *n*—electron transfer number, *F*—Faraday’s constant (96,485 C/mol).

**Figure 5:**
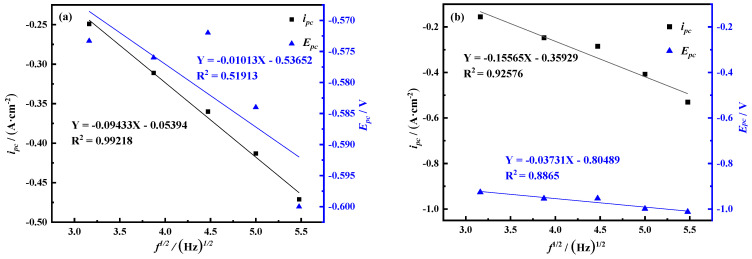
Curves of reduced peaks *i_pc_*-*f*^1/2^ and *E_pc_*-*f*^1/2^ (**a**) peak C (**b**) peak B.

Generally speaking, this method is only effective for reversible reactions, and the reversibility can be judged by the linear relationship between the square root of frequency and the peak current density. Draw the relationship between the peak current density and the square root of frequency according to the square wave volt-ampere data in Figure 4, as shown in Figure 5. It can be seen from the figure that for peak B, *i_pc_*, *E_pc_* and *f*^1/2^ are linear, but for peak C, *E_pc_* and *f*^1/2^ are not linear. At the same time, it is judged that the electrochemical reduction process corresponding to this peak is irreversible, so the number of transferred electrons can be calculated for peak B by Formula (1).

**Figure 6:**
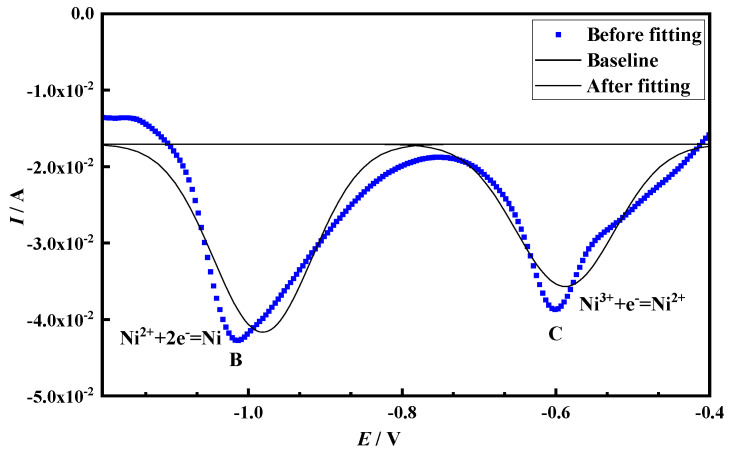
Peak shape fitting diagram of square wave voltammogram of LiNiO_2_ in NaCl-CaCl_2_ molten salt under 30 Hz condition.

Peak B was fitted according to the square wave voltammetry, as shown in Figure 6. The half-width of peak B is *W*_1/2_ = 0.15, and the number of electrons transferred from peak B is *n* = 2.06, calculated from Formula (1), which is about equal to *n* = 2, and the number of electrons transferred from peak C is judged to be 1. Therefore, the reaction of Ni^3+^ on the Mo electrode is shown in Equations (2) and (3):

Peak A: Ni^3+^ + e^−^ = Ni^2+^ (2)

Peak B: Ni^2+^ + e^−^ = Ni (3)

It can be seen from the above analysis that the electrochemical reduction process of Ni^3+^ on the Mo electrode is divided into two steps, which is consistent with the results of the cyclic voltammetry curve test.

#### 3.1.3. Chronoamperometry

**Figure 7:**
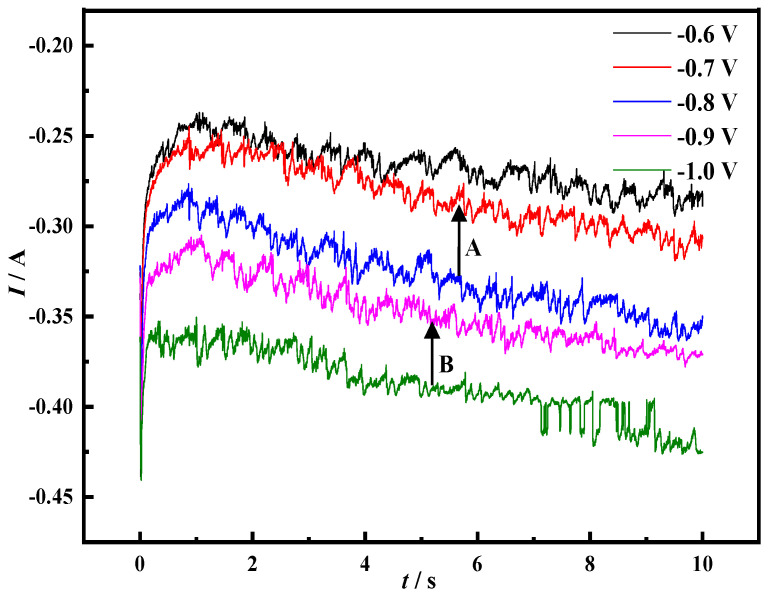
Cyclic voltammetry curve of Mo electrode in NaCl-CaCl_2_-LiNiO_2_ molten salt system (scanning speed: −0.6–−1.0 V/s, *T* = 750 °C, reference electrode: Pt electrode).

Figure 7 shows the *I-t* curve of the Mo electrode in the molten salt system of NaCl-CaCl_2_-LiNiO_2_ at 750 °C under different potential tests. Due to the electric double layer charging behavior between the electrode and the electrolyte, the initial current value in the figure is relatively large. Under different potential conditions, the initial current is proportional to the potential.

In the potential range of −0.6–−0.7 V, no obvious potential transition occurs. The current step A at −0.8 V indicates that Ni^3+^ has electrochemical reduction behavior in the potential range of −0.7–−0.8 V, corresponding to the reduction step of Ni^3+^ → Ni^2+^. With the change in potential, the current step B at −1.0 V indicates that Ni^2+^ has electrochemical reduction behavior in the range of −0.9–−1.0 V, corresponding to the reduction step of Ni^2+^ → Ni.

#### 3.1.4. Open Circuit Chronopotentiometry

**Figure 8:**
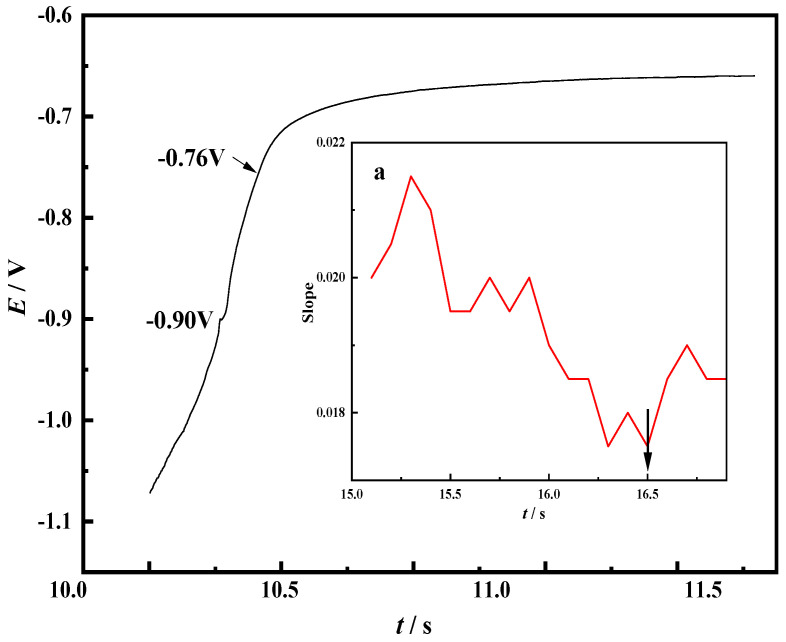
Open circuit chronopotential curve of Mo electrode in NaCl-CaCl_2_-LiNiO_2_ molten salt system (**a**) Slope graph at different times (*T* = 750 °C, reference electrode: Pt electrode).

In order to further study the electrochemical reduction behavior of Ni^3+^ on the Mo electrode in the NaCl-CaCl_2_-LiNiO_2_ molten salt system, the open-circuit chronopotentiometry was used for determination. This method records the potential difference between the working electrode and the reference electrode after the electrode is charged for a short period of time, and the result is shown in Figure 8.

According to the chronocurrent curve shown in Figure 7, the potential corresponding to the two-step oxidation process of nickel can be roughly judged. In the chronopotential curve shown in Figure 8, the plateau corresponding to −0.9 V is more obvious. For differentiation, consider part of the data in the figure. The result is shown in Figure 8a. When *t* = 16.5, the slope is the smallest, indicating that the curve has reached the smoothest stage, and the corresponding potential is −0.76 V. This process is consistent with the appearance mechanism of peak A in the cyclic voltammetry curve. The plateau near −0.90 V exists for a short time, indicating that as the potential increases negatively, the reduction rate increases, and the Ni^2+^ concentration in the molten salt decreases with the rapid progress of the reaction. This process is consistent with the appearance mechanism of peak B in the cyclic voltammetry curve. According to the above analysis, in the NaCl-CaCl_2_-LiNiO_2_ molten salt system, the electrochemical reduction process of Ni^2+^ on the Mo electrode is Ni^3+^ → Ni^2+^ → Ni.

### 3.2. The Electrochemical Behavior of LiNO_2_ in the Solid Electrode on the Mo Cavity Electrode

#### Cyclic Voltammetry

**Figure 9:**
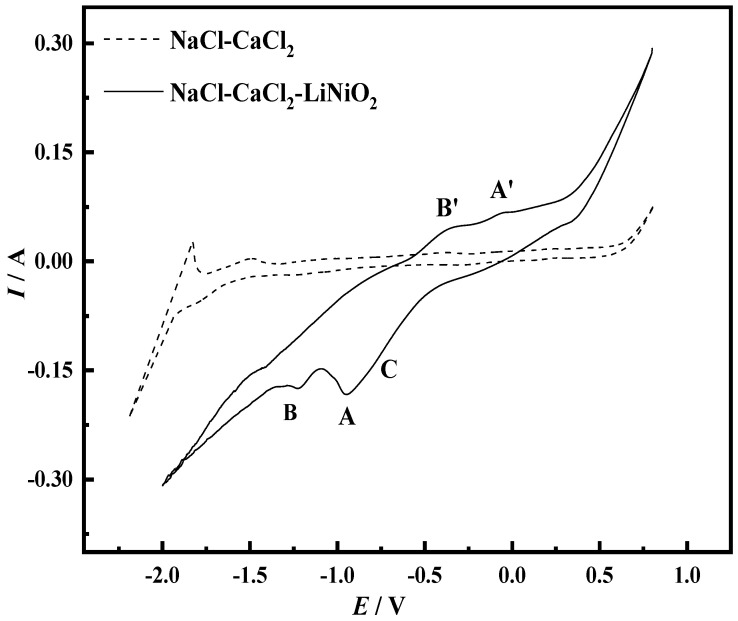
Cyclic voltammetry diagram of Mo cavity electrode filled with or without LiNiO_2_ in NaCl-CaCl_2_ molten salt (scanning speed: 0.2 V/s, *T* = 750 °C, reference electrode: Pt electrode).

At a scanning speed of 0.2 V/s, a cyclic voltammetry test was carried out on the Mo cavity electrode filled with or without LiNiO_2_ in the NaCl-CaCl_2_ molten salt system, and the results are shown in Figure 9. In the NaCl-CaCl_2_ molten salt system, there is no oxidation–reduction reaction in the 1.69–0.57 V potential range, which can be used as the potential scanning range of the Mo cavity electrode filled with LiNiO_2_. There are three peaks, A, B and C, in the curve, shown by the solid line. From the previous analysis, it is known that peak C is the alloy peak formed by Ni and Mo, so there is a two-step electrochemical reduction process for Ni^3+^ on the Mo electrode.

**Figure 10:**
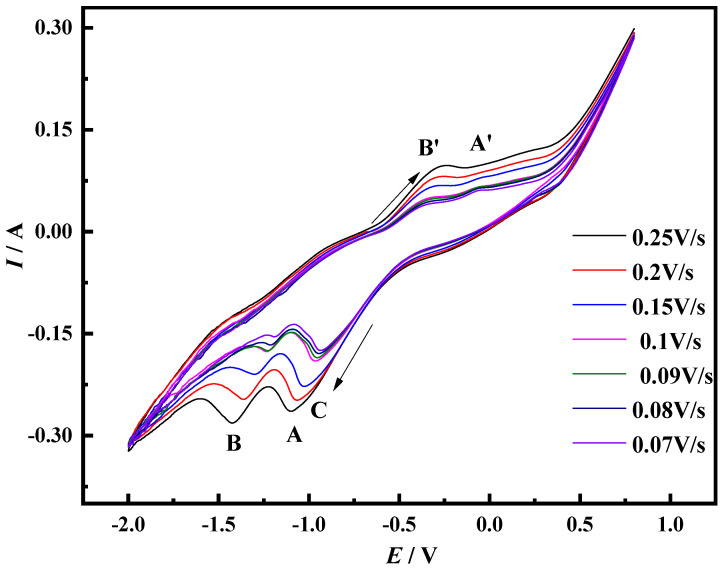
CV diagram of Mo-hole electrode in NaCl-CaCl_2_ molten salt system after filling LiNiO_2_ (scanning speed: 0.07–0.25 V/s, *T* = 750 °C, reference electrode: Pt electrode).

At 750 °C, cyclic voltammetry was performed on the Mo cavity electrode filled with LiNiO_2_ at different sweep speeds in the NaCl-CaCl_2_ molten salt system. The results are shown in Figure 10.

LiNiO_2_ belongs to an ionic lattice structure, and the ions on each node in the powder crystal vibrate around their respective equilibrium positions. At 750 °C, as the applied potential increases, the ion vibration that obtains energy increases. When the potential reaches a certain condition, O^2−^ obtains the activation energy required for displacement, overcomes the binding of surrounding ions, and diffuses to the anode through the molten salt [25]. The exposed Ni^3+^ maintains the equilibrium of the valence state through an electrochemical reduction reaction. When the reduction potential of Ni^3+^ → Ni^2+^ is reached, the current increases with the increase of the potential, and the process is mainly controlled by the interface charge transfer step. With the negative shift in the scanning potential, O^2−^ continuously ionizes from LiNiO_2_ and concentrates near the cathode interface. When the applied potential reaches the peak potential, the concentration of the reactant on the surface of the cathode is maximized to form a concentration polarization phenomenon, which causes the peak potential to shift. The diffusion process cannot be maintained in a stable state so that the electro-chemical reduction rate is not equal to the diffusion rate of O^2−^, the thickness of the diffusion layer increases and the concentration gradient decreases. Therefore, the diffusion current gradually decreases, and finally peak A is formed. When the next reduction potential is reached, the formation of the reduction peak B still complies with the above theoretical analysis. When scanning in the forward direction, the Ni elemental substance formed during the reduction process is oxidized again, and each reduction process corresponds to peak A′ and peak B′ in Figure 10. Under faster scanning speed, the electro-chemical reduction time is shorter, and O^2−^ ionized from the LiNiO_2_ matrix accumulates at the cathode interface, forming a thinner transient diffusion layer and a larger concentration gradient. The above phenomenon leads to an increase in current and polarization. Under slower sweep conditions, the long electrochemical reduction time allows O^2−^ to fully transport at the electrode/molten salt interface to form a thicker transient diffusion layer and a smaller concentration gradient. The above electrochemical behavior leads to the reduction in the oxidation peak and the reduction peak.

In addition, with the increase in the scan rate, the peak potentials of peaks A and B continue to move in the negative direction, and there is no obvious oxidation peak corresponding to peak A in the figure. This phenomenon shows that the reduction is gradually turning in an irreversible direction. According to the above analysis, it is judged that peak A corresponds to an irreversible reduction process, and peak B corresponds to a quasi-reversible reduction process.

**Figure 11:**
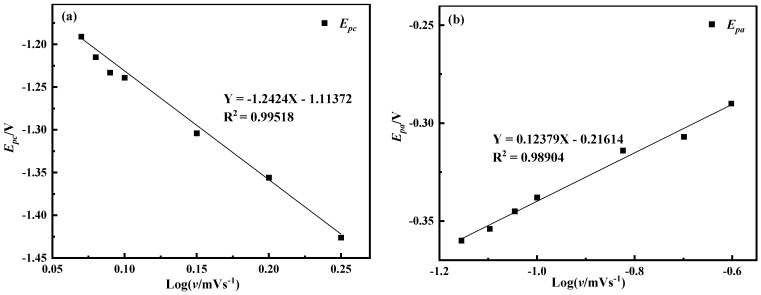
The log*v*-*E_pc_* and log*v*-*E_pa_* curves of peak B (A = 0.32 cm^2^) (**a**) log*v*-*E_pc_* (**b**) log*v*-*E_pa_*.

The curves of log*v*-*E_pc_* and log*v*-*E_pa_* of peak B are plotted respectively according to Figure 10, as shown in Figure 11. The binary primary equations of diffusion coefficient and the number of transferred electrons are listed according to the slope, and the deduced Equations (4) and (5) combined with the primary binary equations are used to calculate the number of transferred electrons at peak B.

*E*_pa_ = *E*° (*RT*/*αnF*) ln(*RTk*°/*αnF*) + (*RT*/*αnF*) ln*v* (4)

*E*_pc_ = *E*° (*RT*/*αnF*) ln(*RTk*°/(1 − *α*)*nF*) + (*RT*/(1 − *α*)*nF*) ln*v* (5)

According to Figure 11, the linear equation of *E_pc_* and log*v* can be obtained as Equation (6), and the linear equation of *E_pa_* and log*v* is Equation (7).

*y* = −1.2424*x* – 1.11372 (6)

*y* = 0.12379*x* – 0.21614 (7)

In the formula: *R*—molar gas constant (8.3145 J/mol/K), *T*—temperature (K), *n*—electron transfer number, *F*—Faraday’s constant (96,485 C/mol).

**Figure 12:**
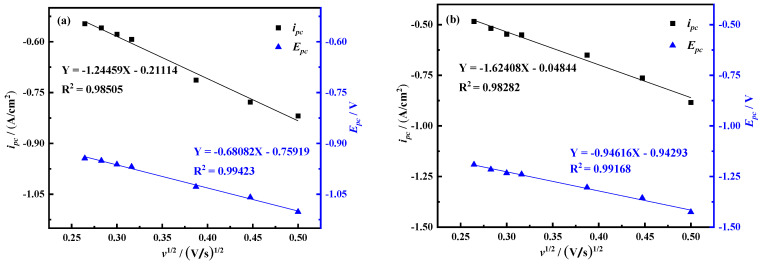
The *i_pc_*-*v*^1/2^ and *E_pc_*-*v*^1/2^ curves of reduction peaks A and B (**a**) peaks A (**b**) peaks B.

The solution shows that *n* = 1.8, which is approximately equal to 2. Therefore, the number of electrons transferred in the electrochemical reduction reaction corresponding to peak B should be 2. In summary, Ni^3+^ reduction is divided into two steps, namely Ni^3+^ + e^−^ = Ni^2+^, Ni^2+^ + 2e^−^ = Ni. The *i_pc_*-*E_pc_*-*v*^1/2^ curves of peaks A and B were respectively drawn according to Figure 10, as shown in Figure 12. It can be seen from the figure that the *i_pc_* and *v*^1/2^ of the two have a linear relationship. It can be judged that the reduction in Ni^3+^ to Ni^2+^ is controlled by the electron transfer step, and the reduction in Ni^2+^ to Ni is controlled by the electron transfer step and the diffusion process.

### 3.3. Preparation of Nickel by Electrolysis of LiNiO_2_ with Molten Salt Constant Current

**Figure 13:**
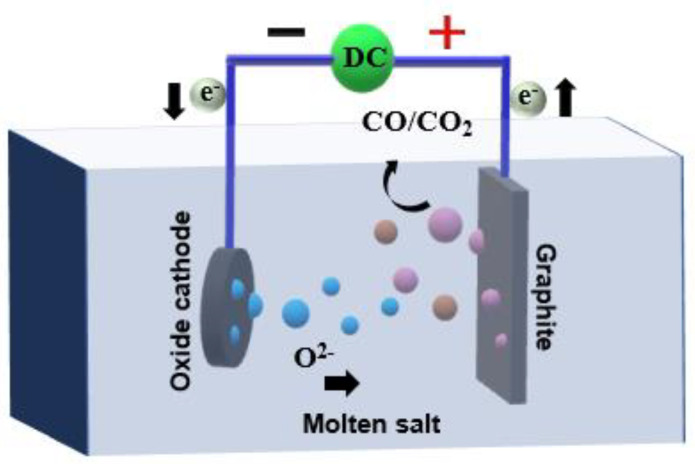
Electrolysis mechanism diagram.

Constant current electrolysis is used to study the reduction process of Ni, and its intermediate products, combined with the reaction process, the interface diffusion and transfer of Ni3+ reduction, are analyzed, and the reduction mechanism of Ni electrolysis is explored. The electrolysis mechanism diagram is shown in Figure 13. I have confirmed.

**Figure 14:**
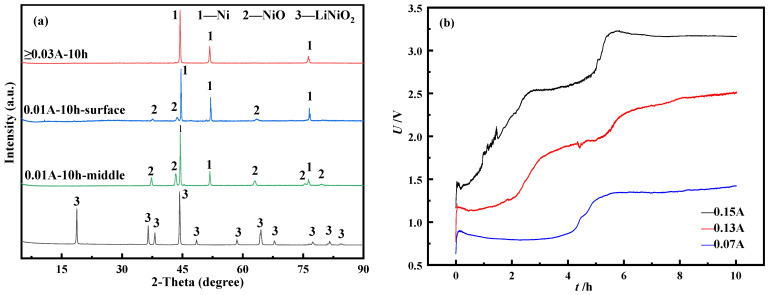
(**a**) XRD patterns of the product after 10 h electrolysis under different current conditions (**b**) *U-t* diagram of electrolysis at 750 °C for 10 h at 0.07–0.15 A.

Under the condition of 750 °C and 10 h of electric deoxidation time, this study selects different currents to recover and prepare metallic nickel. The products of 10 h deoxygenation at different currents are shown in Figure 14a. Under the conditions of 0.01 A and 10 h, Ni and NiO were mainly formed on the surface of the product. Although Ni and NiO also existed inside the product, there was more NiO than the surface. The above phenomenon shows that under this current condition, the electrochemical reaction continuously extends from the outside to the inside along the three-phase interface (3 PIs), and this behavior causes part of the NiO inside the product to fail to continue to be reduced.

In addition, the presence of NiO also proves again that there is a two-step electrochemical reduction behavior of Ni^3+^ in LiNiO_2_. When the current is greater than or equal to 0.03 A, the products after 10 h are all Ni elemental. It can be seen that under the same deoxygenation time condition, a larger current can promote the reduction reaction, so that NiO can continue to be reduced to a Ni elementary substance. It can be seen that under the same deoxygenation time condition, a larger current can promote the reduction reaction, so that NiO can continue to be reduced to a Ni elementary substance. It can be seen from Figure 14b that different currents cause differences in the degree of electron accumulation at the electrodes, resulting in different starting positions of the curve. In addition, comparing the time range corresponding to each reaction platform on the three curves in the figure, the first platform in the *U*-*t* curve measured under the condition of 0.07 A current corresponds to about 4 h, while the second platform corresponds to the duration of “shorter”. The above phenomenon shows that in the first electrochemical reduction process, more NiO and a small amount of Ni element are mainly formed. Therefore, in the second reduction process, the Ni elementary substance is formed faster. The current condition of 0.15 A promotes the progress of electrochemical reduction, so that the reduction reaction is concentrated in the second step of the reaction process. Under the condition of 0.13 A, the whole reaction process is relatively stable, and there is a good transition time. Under the conditions of an electrolysis time of 10 h and currents of 0.07 A, 0.13 A and 0.15 A, this study calculates the actual charge and the theoretical charge according to Equations (8) and (9), and finally calculates the current efficiency of the three, as shown in the Table 1 shown.

*Q* = *It* (8)

*Q* = *nzF* (9)

In the formula, *n* is the amount of substance in the product under theoretical conditions, *z* is the number of transferred electrons, *t* is time (s), and *F* is Faraday’s constant (96,485 C/mol).

**Figure 15:**
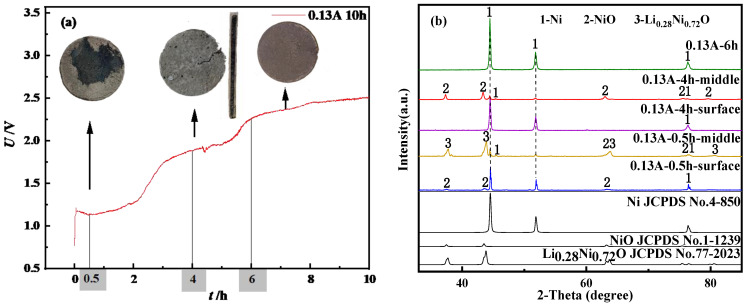
(**a**) 0.13 A *U-t* curve of 10 h electrolysis (**b**) 0.13 A XRD detection of deoxidation products at different times.

It can be seen from the table that as the current increases, the current efficiency decreases. This situation is because the increase in current causes the actual charge to increase, while the theoretical charge has a relatively small change, which eventually leads to a gradual decrease in current efficiency. At 0.13 A and 750 °C, 0.7 g lithium nickelate was subjected to constant current deoxygenation studies for 0.5 h, 4 h and 6 h, respectively. Further study on the influence of time on the electrochemical reduction process through product analysis is needed. The result is shown in Figure 15.

**Figure 16:**
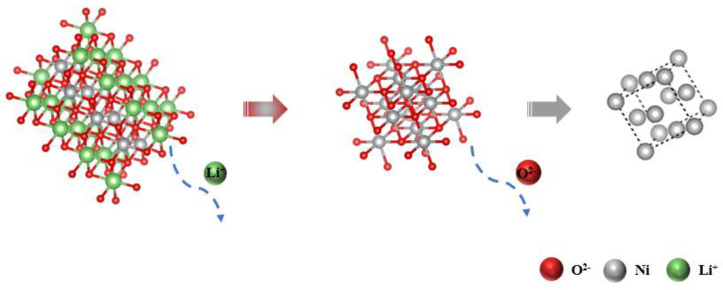
LiNiO_2_ atomic structure change during electrolysis.

The *U*-*t* curve is shown in Figure 15a. In the reduction process of Ni^3+^, different reduction stages correspond to different potential platforms. According to the XRD pattern of the 0.5 h product shown in Figure 15b, it can be seen that the inside of the particles is mainly Ni and NiO, and most of the intermediate products Li_0.28_Ni_0.72_O, which is only caused by the slow extension of the three-phase interface during the reduction process. At this time, the black product inside the product is NiO and Li_0.28_Ni_0.72_O, and the steel gray product appears at the three-phase interface, which is metallic Ni. As the reaction progresses, O^2−^ continuously ionizes from the LiNiO_2_ matrix and concentrates near the cathode. At the same time, the produced Ni element adheres to the surface of the substrate as a conductor and further hinders the ionization of O^2−^ and the transfer of electrons. The above phenomena cause electrons to accumulate in the electric double layer, and eventually lead to a negative increase in potential. When the second step reduction potential is reached, a second platform corresponding to 4 h is formed in the figure. Combined with the XRD test results, it can be seen that the surface of the 4 h product is mainly Ni, and the internal components are mainly Ni and NiO. The black substance inside the product gradually decreases, indicating that, as time goes by, O^2−^ in Li_0.28_Ni_0.72_O is continuously removed and, finally, NiO is formed. This phenomenon also further confirms that Ni^3+^ conducts electricity from the outside to the inside of the electrode surface along the three-phase interface. When the time was increased to 6 h, the NiO inside the product was completely deoxidized, and Ni^2+^ was reduced to elemental Ni. As time continues to extend, there are no other oxidation–reduction reactions in the molten salt, and the potential ultimately remains in a relatively stable state. The atomic structure diagram is shown in Figure 16.

**Figure 17:**
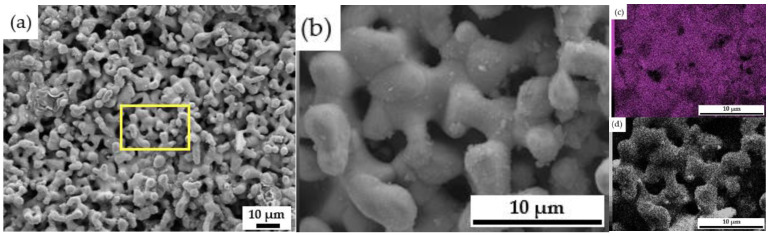
SEM images of electrolyzed products at 1000 times (**a**) and 5000 times (**b**) after 10 h, as well as EDS and surface scan maps at 5000 times ((**c**): Ni element (**d**): O element).

Figure 17 is the SEM, EDS and surface scan detection spectrum of the 10 h electrolysis product under different multiple conditions. It can be seen that the metallic nickel has a honeycomb skeleton structure, and its content reaches 96.39%. The electrolytic metallic nickel is silver-white with metallic luster and magnetic.

**Figure 18:**
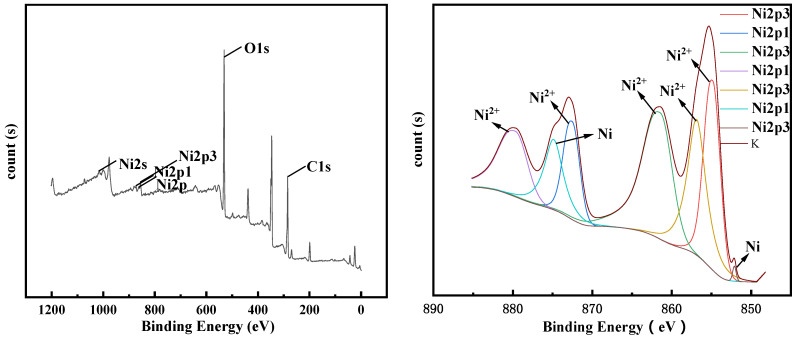
XPS test results of the product after 0.13 A electrolysis for 4 h.

**Figure 19:**
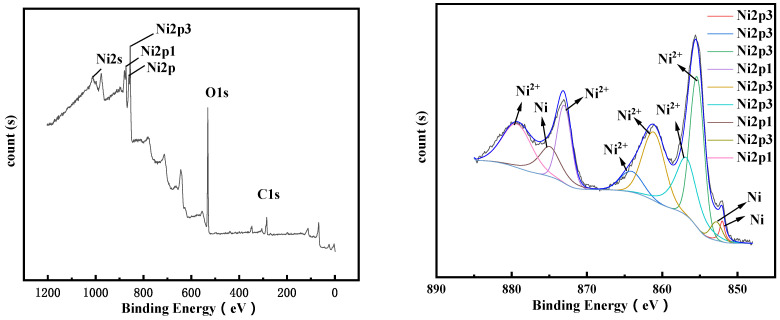
XPS test results of the product after 0.13 A electrolysis for 10 h.

XPS detection was performed on the electrolysis products after 4 h and 10 h, and the results are shown in Figure 18 and Figure 19, respectively. It can be seen from the XPS spectrum that elemental nickel appears in the product of electrolysis at 4 h. As the electrolysis time increases, more Ni^2+^ is reduced to elemental Ni, and the peaks corresponding to elemental Ni in the XPS spectrum increase. Compared with the XPS spectrum after 4 h of electrolysis, the 2p peak of Ni after 10 h of electrolysis is weaker and wider, while the 2p peak of part of Ni^2+^ is strengthened. This phenomenon is caused by the increase in oxides on the surface of the sample [26].

### 3.4. The Behavior of Lithium under Constant Current Conditions

LiNiO_2_ belongs to the trigonal crystal system. The oxygen atoms at position 6c are closely packed in cubic form to form a crystal framework, which occupy positions 3a and 3b, respectively. Li atoms and Ni atoms are located in alternate octahedral positions in the framework, with obvious layered structure, and the space group is R/3m. Due to the special electronic arrangement of Ni^2+^, part of Ni^2+^ existing in LiNiO_2_ will occupy the position of Ni^3+^. In order to maintain charge balance and the similar atomic radii of Ni^2+^ and Li^+^, the position of Li^+^ will also be occupied by Ni^2+^, and at the same time, during the electrolysis process, Ni^2+^ will transform to Ni^3+^ with a smaller radius, causing the structure to change, and the position of Li^+^ will further lose the ability to insert lithium [27,28]. In addition, under the layered structure, van der Waals force and ionic bonds are used to maintain the interlayer and the inner layer, respectively. Because the van der Waals force is weak, the Li^+^ in the layer is required to maintain the stability of the structure through electrostatic action. However, there is a big difference between the Li^+^(1s^2^) energy level and the O^2−^ energy level, while the Ni^3+^(3d^7^) and O^2−^(2p^6^) energy levels are relatively similar, which makes the electron cloud overlap between Li-O more than that between Ni–O. If it was smaller, the Li-O bond energy would eventually be weaker. Therefore, the Li–O bond will be preferentially broken during the electrolysis process, and Li^+^ will escape from the Ni-O layer [29].

In order to further study the electrochemical behavior of Li^+^ in molten salt or Li^+^ in cathode reduction particles, thermodynamic calculations of related reactions are carried out. Equation (10) shows that the Gibbs free energy of the reaction is negative. Therefore, the reduction in Li^+^ will proceed with more difficulty. During the electrochemical scanning process, the redox peak of Li did not appear, which is consistent with the analysis of the cyclic voltammogram.

4Li + O_2_ = 2Li_2_O Δ*G*^Θ^ = −923.712 kJ/mol (10)

**Figure 20:**
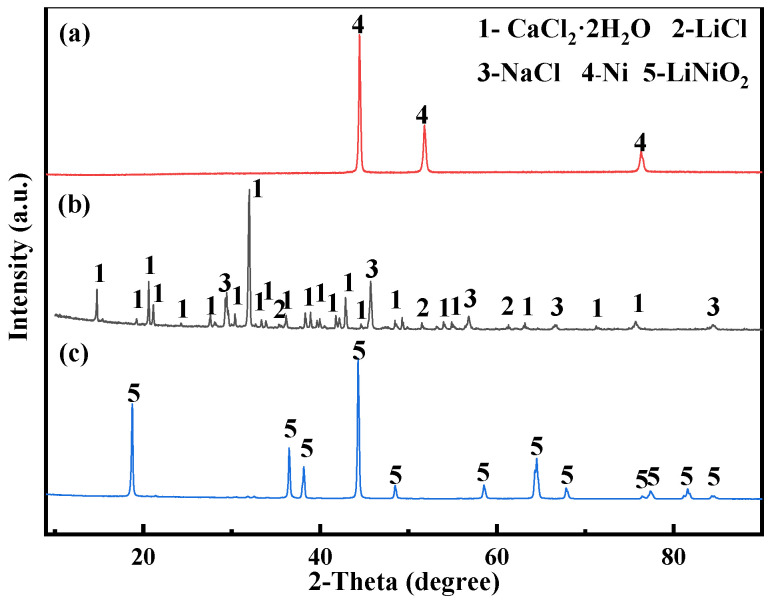
(**a**) XRD pattern of the product after 10 h of 0.18 A constant current electrolysis (**b**) XRD pattern of molten salt near cathode after electrolysis (*T* = 750 °C) (**c**) XRD analysis of LiNiO_2_ in molten salt.

In this study, 0.7 g of LiNiO_2_ powder was made into the cathode and smooth graphite flakes were used as the anode, and they were put into NaCl-CaCl_2_ molten salt and electrolyzed at 750 °C with a constant current of 0.18 A for 10 h. After the electrolysis, XRD analysis was performed on the molten salt and the product near the cathode, and the result is shown in Figure 20. It can be seen from Figure 20 that the product is elemental Ni after electrolysis for 10 h under the condition of 0.18 A current. After electrolysis, there is LiCl in the XRD of the molten salt near the cathode. The above phenomenon shows that Li^+^ in LiNiO_2_ finally enters the molten salt. The constant cell voltage electrolysis in the two-electrode system further supports the conclusions obtained by cyclic voltammetry, square wave voltammetry, open circuit potential and chronoamperometry.

## 4. Conclusions

The electrochemical reduction behavior of LiNiO_2_ in NaCl-CaCl_2_ molten salt (molar ratio: 1:1) and on the Mo cavity electrode was investigated by cyclic voltammetry, square wave voltammetry, chronoamperometry and open-circuit potential at 750 °C. Through the method of constant current electrolysis, the process of preparing metal nickel by reduction of lithium nickelate was analyzed, and the following conclusions drawn:

(1) The electrochemical reduction process of Ni^3+^ in the two research systems is an irreversible–quasi-reversible reaction process, and the electro-chemical reduction process is divided into two steps. There are alloy peaks formed by Ni and Mo in the cyclic voltammetry curves of the two systems.
(2) Under 10 h, 0.01–0.15 A current conditions, the material reduction process is LiNiO_2_ → NiO → Ni. When the current is 0.01 A, the product is elemental Ni and a small amount of NiO. When the current is not less than 0.03 A, the product is mainly metallic Ni. In the whole process, under the conditions of 0.07 A and 10 h of electrolysis, the highest current efficiency is 77.9%.
(3) At 0.13 A and 0.5 h after electrolysis, the intermediate product Li_0.28_Ni_0.72_O, produced in a short time, is chemically dissolved in the molten salt, and finally deoxidized to produce NiO. After the electrolysis time was extended to 6 h, the product was metallic Ni. The presence of LiCl in the molten salt near the cathode after electrolysis indicates that Li^+^ has not been reduced during the entire reaction, but entered the molten salt in the form of ions.

## Figures and Tables

**Table 1:** 10 h electrolysis efficiency at 750 °C at 0.07–0.15 A.

Electrolysis Conditions	LiNiO_2_/g	Current Efficiency/%
0.07 A, 10 h	0.665	77.9
0.13 A, 10 h	0.670	42.2
0.15 A, 10 h	0.668	36.5

## Data Availability

Data sharing is not applicable to this article.

## References

[1] Li Z.Q., Zhuang X.N., Song X.L., Li F., Li Y.S., Gu W.H., Bai J.F. (2021). Research progress on recovery of the cathode material from spent lithium-ion batteries by pyrometallurgy. Environ. Eng..

[2] Hao T., Zhang Y.J., Dong P., Liang F., Duan J.G., Meng Q., Xu B. (2018). Review on recycling cathode materials of spent ternary power lithium-ion batteries. Bull. Chin. Ceram. Soc..

[3] Linda G. (2014). The future of automotive lithium-ion battery recycling: Charting a sustainable course. Sustain. Mater. Technol..

[4] Chen X.P., Ma H.R., Luo C.B., Zhou T. (2017). Recovery of valuable metals from waste cathode materials of spent lithium-ion batteries using mild phosphoric acid. J. Hazard. Mater..

[5] Cou H.P., Pei Z.Y., Zhou G.Z., Liu C., Lv D., Chen X.G., Yu Y., Li M.C. (2019). Study on recycle of the spent ternary Li-ion battery by pyrometallurgical process. China Nonferr. Metal..

[6] Guo M.M., Xi X.L., Zhang Y.H., Yu S.W., Long X.L., Jiang Z.K., Nie Z.R., Xu K.H. (2020). Recovering valuable metals from waste ternary cathode materials of power battery by combined high temperature hydrogen reduction and hydrometallurgy. Chin. J. Nonferr. Met..

[7] Hu J.T., Zhang J.L., Li H.X., Chen Y.Q., Wang C.G. (2017). A promising approach for the recovery of high value-added metals from spent lithium-ion batteries. J. Power Sources.

[8] Lupi C., Pasquali M. (2003). Electrolytic nickel recovery from lithium-ion batteries. Miner. Eng..

[9] Sun M.C., Ye H., Chen W.J., Li H.Y., Xiao J. (2019). Study on Recovering Valuable Metals from Spent Lithium-ion Batteries. Nonferr. Metal. Extr. Metal..

[10] Li X.Y. (2019). Research on Key Technologies of Partial Porous Externally Pressurized Gas Bearing.

[11] Wang X.F., Kong X.H., Zhao Z.Y. (2001). Recovery of noble metal in lithium ion battery. Battery. Bimon..

[12] Li L., Fan E., Guan Y., Zhang X., Chen R. (2017). Sustainable recovery of cathode materials from spent lithium-ion batteries using lactic acid leaching system. ACS Sustain. Chem. Eng..

[13] Zhang H.J., Cheng J.H., Zhu C., Yang J., Gu M. (2019). Recovery of Copper, Cobalt and Nickel from Spent Lithium Ion Batteries by a Combined Process of Acid Leaching and Bioleaching. Hydrometall. China.

[14] Chen G.Z., Fray D.J., Farthing T.W. (2010). Direct electrochemical reduction of titanium dioxide to titanium in molten calcium chloride. Cheminform.

[15] Zhu Y., Meng M.A., Wang D. (2006). Electrolytic reduction of mixed solid oxides in molten salts for energy efficient production of the TiNi alloy. Chin. Sci. Bull..

[16] Qiu G., Wang D., Meng M., Jin X., Chen G.Z. (2006). Electrolytic synthesis of TbFe_2_ from Tb_4_O_7_ and Fe_2_O_3_ powders in molten CaCl_2_. J. Electroanal. Chem..

[17] Peng J., Chen H., Jin X., Wang T., Wang D., Chen G.Z. (2009). Phase-Tunable Fabrication of Consolidated (α+β)-TiZr Alloys for Biomedical Applications through Molten Salt Electrolysis of Solid Oxides. Chem. Mater..

[18] Chen G.Z., Gordo E., Fray D.J. (2004). Direct electrolytic preparation of chromium powder. Metall. Mater. Trans. B.

[19] Gordo E., Chen G.Z., Fray D.J. (2004). Toward optimisation of electrolytic reduction of solid chromium oxide to chromium powder in molten chloride salts. Electrochim. Acta.

[20] Zhang B., Xie H., Lu B., Chen X., Xing P., Qu J., Song Q., Yin H. (2019). A Green Electrochemical Process to Recover Co and Li from Spent LiCoO_2_-Based Batteries in Molten Salts. ACS Sustain. Chem. Eng..

[21] Chen G.Z., Fray D.J. Understanding the electro-reduction of metal oxides in molten salts. Proceedings of the Symposium of Recent Advances in Non-Ferrous Metals Processing, 2004 TMS Annual Meeting.

[22] Li N., Peng Y., Chen Z., Xiong W., Li S. (2021). Preparation of Mg-Zr Alloys through Direct Electro-deoxidation of MgO-ZrO_2_ in CaCl_2_-NaCl Molten Salt. Electrochim. Acta.

[23] Konishi H., Nohira T., Ito Y. (2002). Formation of Dy–Fe alloy films by molten salt electrochemical process. Electrochim. Acta.

[24] Kubota T., Iida T., Nohira T., Ito Y. (2004). Formation and phase control of Co-Gd alloy films by molten salt electrochemical process. J. Alloys Compd..

[25] Ding X. (2015). Study of the Mechanism on Formation of Calcium Ferrite in the Fe_2_O_3_-CaO-SiO_2_ System.

[26] Xu Z.Y. (2019). Study on Grain Orientation and Low Frequency Electromagnetic Properties of FeSiAl Alloy Absorbents. Bachelor’s Thesis.

[27] Pi Q.L. (2009). Study on Surface Coating Modification of Layered Cathodes Materials LiNi_x_Co_1−x_O_2_ for Li-ion Batteries. Master’s Thesis.

[28] Liang L., Li Q., Qiao Q.D., Li P. (2007). Research progress on LiNiO_2_ cathode material for Li-ion batteries. Inorg. Chem. Ind..

[29] Zhang N., Tang Z.Y., Huang Q.H., Han B. (2005). The Synthesis and Modification of Cathode Material LiNiO_2_. Chemistry.

